# Räumlich-epidemiologische Datenanalyse von Pathologiedaten (REDPath): Ein web-basiertes Tool für Onkologie und Versorgungsforschung

**DOI:** 10.1007/s00292-025-01522-x

**Published:** 2025-12-10

**Authors:** Stephanie Strobl, Matthias Martin Gaida

**Affiliations:** 1 Institut für Pathologie, Universitätsmedizin Mainz, Johannes Gutenberg-Universität Mainz, Langenbeckstr. 1, 55131 Mainz, Deutschland; 2 TRON, Translationale Onkologie an der Universitätsmedizin Mainz, Johannes Gutenberg-Universität Mainz, Mainz, Deutschland

**Keywords:** Pathologische Routinedaten, Räumliche Epidemiologie, Geographische Informationssysteme, Onkologische Versorgung, Prävention und Versorgungsforschung, Pathology routine data, Spatial epidemiology, Geographic Information Systems, Oncology care, Prevention and health services research

## Abstract

**Hintergrund:**

In der pathologischen Routinediagnostik entstehen umfangreiche Datensätze, deren Potenzial für räumlich-epidemiologische Analysen – etwa zur Untersuchung von Krankheitsverteilungen, Umweltbelastungen oder Versorgungsstrukturen – bislang ungenutzt bleibt.

**Ziel der Arbeit:**

Mit REDPath (räumlich-epidemiologische Datenanalyse von Pathologiedaten) wurde ein web-basiertes Tool entwickelt, das diese Daten systematisch erschließt. Ziel ist es, Krankheitslasten und Versorgungsrealitäten onkologischer Erkrankungen auf verschiedenen geographischen Ebenen darzustellen und datenbasierte Präventionsstrategien zu unterstützen.

**Material und Methoden:**

Grundlage bilden 41.707 onkologische Diagnosen (ICD-10: C00-C97, 2019–2025) des Instituts für Pathologie Mainz, ergänzt um demographische und umweltbezogene Kontextvariablen. REDPath wurde in C++ und Python programmiert, Visualisierungen erfolgen über Leaflet, statistische Analysen über R (*lme4* und *CARBayes*). Die Daten werden zweistufig (individuell/aggregiert) verarbeitet, um Datenschutz und differenzierte Zugriffsrechte zu gewährleisten.

**Ergebnisse:**

Die REDPath umfasst 3 Module: (1) deskriptive Analysen zur interaktiven Visualisierung von Krankheitsverteilungen, (2) statistische Modelle zur Prüfung geographischer Zusammenhänge und Autokorrelationen sowie (3) ein in Entwicklung befindliches Modul zur Versorgungsforschung, das Einsenderstrukturen darstellt.

**Schlussfolgerung:**

Die REDPath ermöglicht die räumlich-epidemiologische Analyse pathologischer Routinedaten – benutzerfreundlich und ohne statistische Vorkenntnisse. Sein modularer Aufbau erlaubt die Integration weiterer Krankheitsentitäten und Datenquellen und positioniert das Tool damit als Schnittstelle zwischen Pathologie und Epidemiologie mit direktem Nutzen für eine evidenzbasierte Versorgung.

## Abstract (en)

**Background:**

Pathological routine diagnostics generate extensive datasets, yet their potential for spatial epidemiological analyses—for instance for studying disease distributions, environmental exposures, or healthcare structures—has so far remained largely untapped.

**Objective:**

With REDPath (Spatial Epidemiological Data Analysis of Pathology Data), a web-based tool to unlock this data source has been developed. Its goal is to visualize oncological disease burden and healthcare provision across different geographic levels, thereby supporting data-driven prevention and resource allocation strategies.

**Materials and methods:**

The basis consists of 41,707 oncological diagnoses (ICD-10: C00-C97, 2019–2025) from the Institute of Pathology, University Medical Center Mainz, supplemented by demographic and environmental context variables. REDPath was programmed in C++ and Python; visualizations were generated with Leaflet and statistical analyses were performed in R using the *lme4* and *CARBayes* packages. Data were processed on two levels (individual/aggregated) to ensure data protection and differentiated access rights.

**Results:**

REDPath comprises three modules: (1) descriptive analyses for interactive visualization of disease distributions; (2) statistical models to examine spatial relationships and autocorrelations; and (3) a health services module currently in development, visualizing submitting institutions.

**Conclusion:**

REDPath enables spatial epidemiological analysis of routine pathology data—in a user-friendly and accessible manner without statistical expertise. Its modular structure allows for the integration of additional disease entities and data sources, positioning the tool as an interface between pathology and epidemiology, with direct relevance for evidence-based healthcare planning.

In der pathologischen Routinediagnostik entstehen täglich große, gut dokumentierte Datensätze, deren Potential für räumlich-epidemiologische Analysen bislang kaum genutzt wird. Durch die Annotation dieser Daten eröffnen sich neue Möglichkeiten: Geographische Muster in Krankheitsverteilungen können sichtbar gemacht, Zusammenhänge mit Umwelt- und sozioökonomischen Faktoren untersucht und Versorgungsrealitäten bewertet werden. So lassen sich Prävention und medizinische Versorgung gezielter und evidenzbasiert steuern.

Um dieses Potential zu erschließen, wurde REDPath (räumlich-epidemiologische Datenanalyse von Pathologiedaten) entwickelt: ein web-basiertes, benutzerfreundliches Tool, das sowohl deskriptive als auch statistische Analysen von Pathologiedaten ermöglicht, ohne dass Nutzer über spezifisches statistisches Fachwissen verfügen müssen. REDPath erlaubt die Darstellung der Krankheitslast onkologischer Erkrankungen sowie der onkologischen Versorgungssituation auf unterschiedlichen geographischen Ebenen. Es verfolgt dabei zwei Kernziele: die Verknüpfung verschiedener Datenquellen (Routinedaten aus pathologischen Instituten, klinische Daten, Kontextfaktoren) und die Niedrigschwelligkeit in der Anwendung für unterschiedliche Akteursgruppen wie Krebsregister, Krankenkassen oder der öffentliche Gesundheitsdienst.

## Räumlich-epidemiologische Methoden im Überblick

Geographische Informationssysteme (GIS) bilden die technische Grundlage räumlicher Analysen. Sie kombinieren Hard- und Software, um Daten geographisch zu verorten, auf Karten darzustellen und statistisch auszuwerten. Ihre besondere Stärke liegt nicht nur in der Visualisierung von Krankheitsverteilungen, sondern in der Fähigkeit, Gesundheitsdaten mit spezifischen Orten und deren Kontextmerkmalen – etwa sozioökonomischen oder umweltbezogenen Faktoren – zu verknüpfen [17]. Auf diese Weise wird die Interpretation räumlicher Muster erleichtert und Hypothesen zu Risikofaktoren oder Versorgungsdisparitäten können überprüft werden.

Da GIS verschiedene Dimensionen von Gesundheit zusammenführen und Methoden aus Medizin, Geowissenschaften, Statistik und Informatik verbinden, handelt es sich um ein inhärent interdisziplinäres Instrument. Damit nehmen GIS eine zentrale Rolle in der modernen Gesundheitsforschung ein, da sie dazu beitragen komplexe, vielfältige und hochdynamische Gesundheitsdaten darzustellen und zu analysieren.

Räumlich-epidemiologische Analysen lassen sich dabei in drei Hauptansätze einteilen:

- „*disease mapping*“ – die reine Visualisierung von Krankheitsverteilungen.
- *Cluster- und Hotspot-Analysen* – die Identifikation auffälliger Häufungen von Krankheitsfällen.
- *geographische Korrelationsstudien* – die Analyse von Zusammenhängen zwischen Erkrankungen und sozialen, demografischen oder umweltbedingten Variablen.

Gerade für onkologische Erkrankungen sind „disease mapping“ und Korrelationsstudien besonders geeignet, da sie nicht nur geographisch regionale Unterschiede der Krankheitslast sichtbar machen, sondern auch Zusammenhänge zwischen Risikofaktoren und Tumorerkrankungen modellieren können. Visualisierungen der Patientenverteilung im Verhältnis zu Screening- und Behandlungseinrichtungen ermöglichen zudem, mögliche Versorgungslücken oder Überlastungen einzelner Strukturen zu erkennen [17]. Dies kann sich beispielsweise in einer überproportionalen Fallbelastung einzelner Kliniken, einem Mangel an spezialisierten Behandlungseinheiten oder in langen Anfahrtswegen für Patientinnen und Patienten äußern.

## Räumlich-epidemiologische Studien in der Onkologie

Im internationalen Kontext existiert eine Vielzahl von anerkannten Studien, die das Potenzial räumlich-epidemiologischer Methoden in der Onkologie unterstreichen. So wurden etwa deutliche Hotspots für kolorektale Karzinome in den USA über einen Zeitraum von mehreren Jahrzehnten hinweg identifiziert [18], ebenso wie eine Häufung von kolorektalen, Lungen‑, Mamma- und Prostatakarzinomen in aktuellen Querschnittsdaten auf Ebene von Counties (vgl. mit Landkreisen in Deutschland; [11]). Georäumliche Analysen werden dabei nicht nur zur Hotspot-Erkennung eingesetzt, sondern auch, um ungeklärte Krankheitsrisiken aus Fall-Kontroll-Studien durch die Einbeziehung der Dimensionen Ort und Zeit besser zu verstehen, wie beispielsweise bei Untersuchungen zu Clustern von Harnblasenkarzinomen in Südost-Michigan gezeigt wurde [9].

Darüber hinaus dienen räumlich-epidemiologische Methoden der Analyse von Versorgungsstrukturen. So konnten Studien georäumliche Unterschiede in der Mammakarzinombehandlung in den USA nachweisen, bei denen regionale Faktoren einen stärkeren Einfluss auf die Fallzahlen hatten als individuelle Patientenmerkmale [8]. Weitere Arbeiten belegen den Einfluss von Versorgungszugang, sozioökonomischem Status und gesundheitsbezogenen Verhaltensweisen auf die Krebssterblichkeit [7]. Studien zu Zervixkarzinomen in Texas oder zur Krebsversorgung in New Jersey verdeutlichen zudem, wie stark Präventions- und Versorgungsungleichheiten mit sozialen und demographischen Faktoren verknüpft sind [19]. Solche Analysen erlauben nicht nur die Identifikation von Regionen mit besonders hohem Präventionsbedarf, sondern auch von Bevölkerungsgruppen, die durch bestehende Versorgungsangebote bislang nicht erreicht werden und daher von gezielten, angepassten Maßnahmen profitieren könnten.

## Technische Entwicklungen und existierende Tools

Parallel zu dieser studienbasierten Forschung haben sich in den letzten Jahren auch die technischen Möglichkeiten erheblich erweitert. Open-source-Tools wie QGIS (QGIS Foundation) oder kommerzielle Lösungen wie ArcGIS (Esri) erlauben heute die Erstellung interaktiver, datengetriebener Kartenanalysen ohne eigene Programmierung.

Zudem entstanden erste öffentlich zugängliche Plattformen, die Krebsdaten aufbereiten, beispielsweise die *US Cancer Statistics Data Visualizations* der Centers for Disease Control and Prevention (CDC; [21]) oder die *Environmental Facilities and Cancer Map* des New York State Department of Health [12]. Ein weiterer Ansatz ist das Intelligent Analysis Tool (iCAT) von Fadiel et al., das verschiedene öffentlich verfügbare Datenquellen mit Mortalitätsdaten zu Krebserkrankungen kombiniert und interaktive Filter- und Analysemöglichkeiten bereitstellt, um regionale Unterschiede in Krebssterblichkeit zu quantifizieren [4]. Die Anwendung ermöglicht es beispielsweise, den Zusammenhang zwischen sozioökonomischen Faktoren wie Armut oder Zugang zu Gesundheitsversorgung und der Sterblichkeit an spezifischen Tumorentitäten innerhalb definierter Regionen zu untersuchen [4]. Allerdings sind diese Ansätze häufig durch begrenzte Datenvielfalt, mangelnde Aktualität und fehlende Integration relevanter Versorgungsdaten limitiert.

Die hier vorgestellten Anwendungen verdeutlichen, wie sich georäumliche Methoden für die Krebsforschung bereits operationalisieren lassen. Ihre Aussagekraft und Nutzbarkeit bleiben jedoch häufig begrenzt – einerseits durch eingeschränkte Datenvielfalt und fehlende Aktualität, andererseits durch die unzureichende Integration klinischer und versorgungsrelevanter Daten. Bemerkenswert ist, dass die Pathologie in bisherigen Studien und anwendungsorientierten Tools nur selten unmittelbar eingebunden ist, obwohl sie über einen besonders umfangreichen und stetig wachsenden Datenbestand verfügt. Routinedaten aus der pathologischen Diagnostik bilden die Grundlage nahezu jeder onkologischen Versorgung und bieten damit ein enormes, bislang ungenutztes Potenzial für die Versorgungsforschung.

## REDPath – Methodik und Aufbau

Das Projekt REDPath wurde als Machbarkeitsstudie („proof of concept“) konzipiert, basierend auf Methodenentwicklung, Datenintegration und Evaluierung der Nutzbarkeit.

### Datenbasis.

**Abb. 1:**
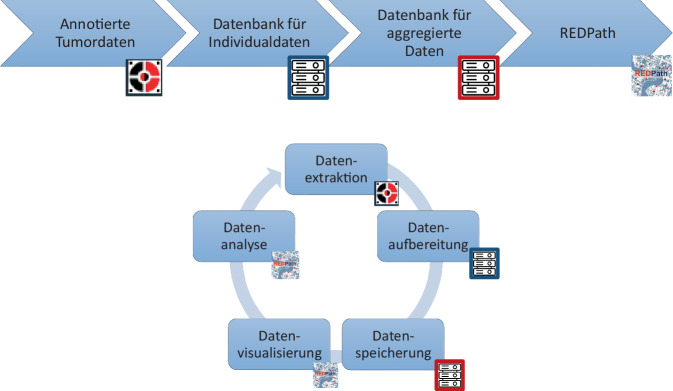
Prozesse der Datenaufbereitung (**a**) und -verarbeitung (**b**)

Grundlage der vorliegenden Arbeit bilden 41.707 onkologische Diagnosen (ICD-10: C00–C97), die zwischen 2019 und August 2025 am Institut für Pathologie der Universitätsmedizin Mainz (im Folgenden: Institut für Pathologie) gestellt wurden. Diese Diagnosen wurden gemäß ICD-O‑3 in der Befundungssoftware PathoPro (ifms) manuell für die Meldung am Krebsregister Rheinland-Pfalz kodiert und automatisiert in eine SQL-basierte Datenbank übertragen. Eine zweite Datenbank aggregiert diese Fälle auf kommunaler Ebene (Stadtteile und Bezirke der Stadt Mainz) und regionaler Ebene (südwestliches Rhein-Main-Gebiet) und ergänzt sie um Kontextvariablen (Umweltfaktoren und demografische Variablen), die durch das Statistikamt der Stadt Mainz zur Verfügung gestellt wurden. Eine genaue Übersicht über die Prozesse, wie Daten aus der Routinediagnostik in die räumlichen Analysen einfließen, findet sich in Abb. 1.

### Zugriff und Datenschutz.

Durch den zweistufigen Aufbau der Datenbankarchitektur können differenzierte Zugriffsrechte umgesetzt werden: Während ärztliches, autorisiertes Personal Zugriff auf pseudonymisierte Einzeldaten erhält (z. B. im Rahmen der Datenaufbereitung), greifen REDPath selbst sowie beteiligte Entwickler ausschließlich auf aggregierte, nicht rückverfolgbare Datenebenen zu. Eine ausführlichere Darstellung des Datenschutzkonzepts findet sich im letzten Abschnitt dieser Arbeit.

### Technische Umsetzung.

Programmiert wurde REDPath in C++ und Python [16], Visualisierungen erfolgten über die JavaScript-Bibliothek Leaflet [1], während die statistischen Analysen über R [15] realisiert wurden. Die Generalized Linear Mixed Models (GLMER) wurden mit dem R‑Paket *lme4* durchgeführt [3], die Intrinsic Conditional AutoRegressive Models (ICAR) mithilfe des Pakets *CARBayes *[10].

## REDPath: Von der Diagnostik zur Versorgung

**Abb. 2:**
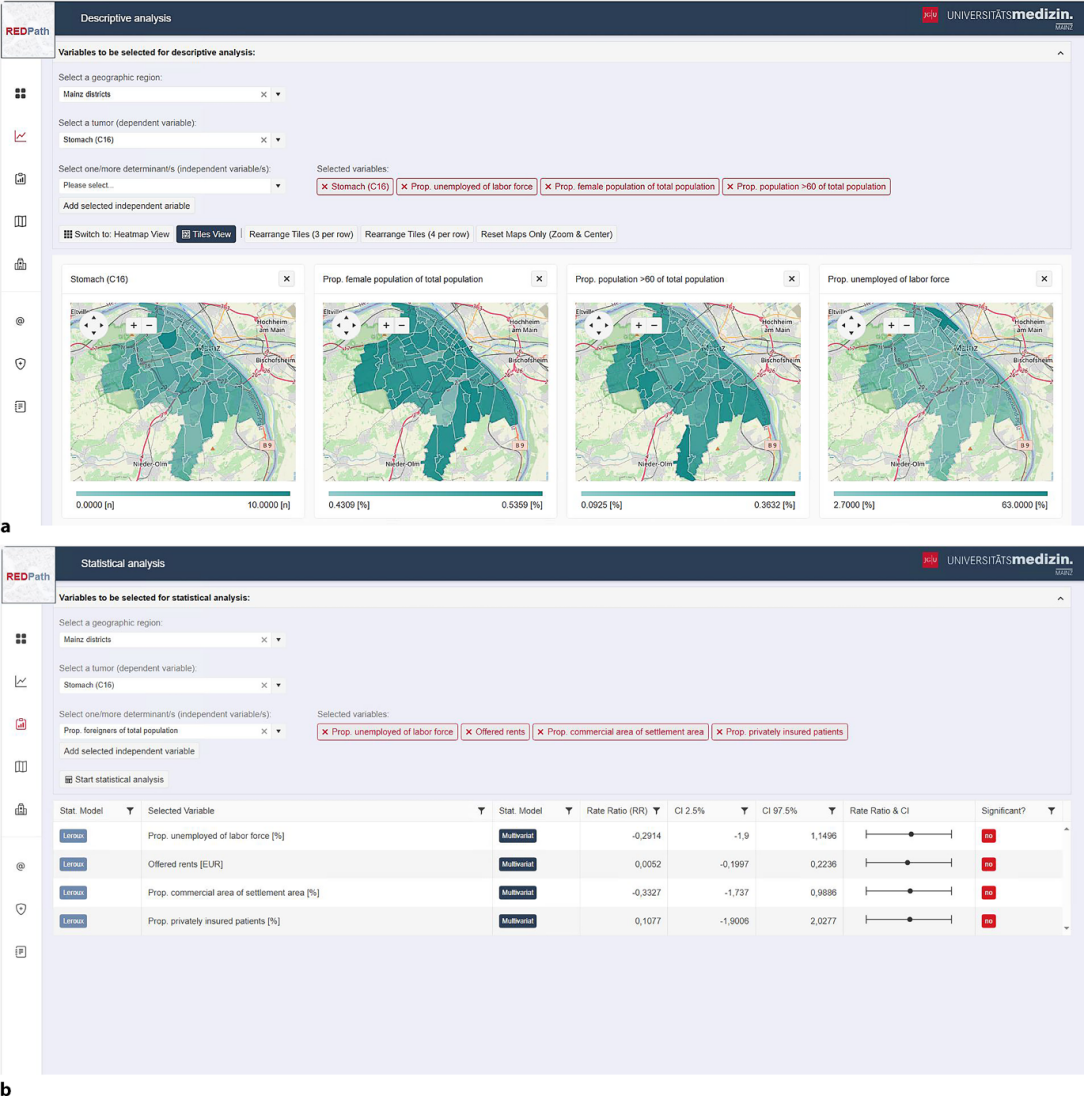
Benutzeroberflächen für die deskriptive (**a**) und statistische (**b**) Analyse in REDPath (räumlich-epidemiologische Datenanalyse von Pathologiedaten)

Ein zentrales Modul von REDPath bilden die *deskriptiven Analysen*, die eine intuitive und zugleich anschauliche Darstellung von Krankheitsverteilungen ermöglichen. Nutzer wählen zuerst die gewünschte geographische Ebene – etwa regionale oder kommunale Einheiten – und legen anschließend eine oder mehrere onkologische Erkrankungen sowie relevante Kontextvariablen fest. Letztere umfassen beispielsweise umweltbezogene oder demographische Faktoren wie den Anteil an Verkehrs- oder Grünflächen, die Bevölkerungsdichte oder die Arbeitslosenquote. Unterhalb dieser drei Auswahlfelder werden die gewählten Variablen jeweils als eigene Kachel mit zugehöriger Legende graphisch dargestellt (Abb. 2a). Die Kacheln lassen sich flexibel anordnen, verschieben und vergrößern, wodurch ein individueller Analysefokus ermöglicht wird. Ziel der deskriptiven Analyse ist es, die Nutzer zunächst mit den vorhandenen Daten vertraut zu machen und gleichzeitig Hypothesen zu potenziellen Zusammenhängen zwischen Erkrankungen und Kontextfaktoren zu generieren. Diese Hypothesen können im Anschluss im Modul für *statistische Analysen* geprüft werden.

Für die statistischen Untersuchungen stehen unterschiedliche räumlich-epidemiologische Modelle zur Verfügung. GLMER dienen der Untersuchung regionaler Determinanten, während ICAR für Szenarien mit räumlicher Autokorrelation eingesetzt werden können. Ob eine solche Autokorrelation vorliegt, wird zuvor über die Moran’s-I-Statistik überprüft. Zeigt sich eine räumliche Abhängigkeit, kommen entsprechend ICAR-Modelle wie BYM oder Léroux zur Anwendung. Die Bedienung des statistischen Moduls ähnelt der deskriptiven Analyse: Zunächst werden geographische Ebene und abhängige Variable gewählt, beispielsweise eine bestimmte Tumorerkrankung. Danach können darunter beliebig viele unabhängige Variablen ergänzt werden, deren Einfluss auf die räumliche Verteilung der abhängigen Variable untersucht werden soll. Die Ergebnisse werden tabellarisch dargestellt und beinhalten die jeweils angewandte Methode, univariate und multivariate Parameter, Effektstärken, Konfidenzintervalle sowie eine einfache Kennzeichnung des Signifikanzniveaus (Abb. 2b).

Bei der Gestaltung der Ausgabe wurde besonderer Wert darauf gelegt, zentrale statistische Kennzahlen vollständig zu erhalten und gleichzeitig eine intuitive Interpretation auch für Nutzer ohne vertiefte Statistikkenntnisse zu ermöglichen. Wichtig ist dabei der Hinweis, dass auf diese Weise zwar Zusammenhänge identifiziert, jedoch keine kausalen Mechanismen nachgewiesen werden können. Ein weiterer wichtiger Hinweis ist, dass die Berücksichtigung der Entfernung zum Studienzentrum fester Bestandteil der Modelle ist. Mit zunehmender Distanz zum Institut für Pathologie sinkt die Wahrscheinlichkeit, dass eine Krebsdiagnose lokal gestellt wird, was die Fallzahlen in peripheren Gebieten verringern und die Ergebnisse verzerren könnte. Dieses Vorgehen wurde bereits am Beispiel von *H.-pylori*-Infektionen und Magenkarzinomen erfolgreich validiert [20]. Gleichzeitig ist zu betonen, dass das Institut für Pathologie der Universitätsmedizin Mainz die einzige pathologische Einrichtung in Mainz ist. Es versorgt damit nicht nur das Universitätsklinikum Mainz, sondern auch zahlreiche regionale Kliniken und ambulante Praxen, wodurch eine sehr hohe Fallabdeckung innerhalb des Studiengebiets gewährleistet ist.

Ein drittes, sich derzeit noch in der Entwicklung befindliches Modul widmet sich der *Versorgungsforschung*. Hierbei stehen verschiedene Anzeige- und Auswerteoptionen zur Verfügung.

**Abb. 3:**
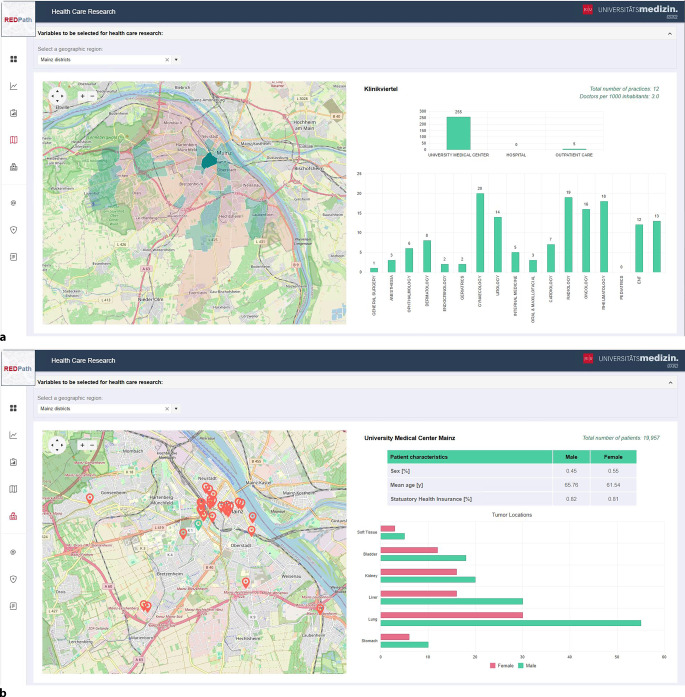
Das Modul Versorgungsforschung in REDPath (räumlich-epidemiologische Datenanalyse von Pathologiedaten), in regionaler (**a**) und individueller (**b**) Ansicht

Auf Flächenebene können Nutzer beispielsweise darstellen, wie viele Einsender es pro geographischer Einheit gibt, wie hoch die Einsenderdichte ausfällt, wie die generelle Versorgungsstruktur aussieht und welcher Fachrichtung die Einsender angehören (Abb. 3a).

Auf Punktmusterebene lassen sich einzelne Einsender auswählen, sodass Informationen zur Anzahl der dort betreuten Krebspatienten, zur demographischen Zusammensetzung sowie zu den behandelten Tumorentitäten sichtbar werden (Abb. 3b).

An der Datengrundlage für dieses Modul wird derzeit noch gearbeitet, sodass es künftig eine wichtige Erweiterung des Funktionsspektrums darstellen wird. Geplant ist beispielsweise die Integration zusätzlicher Variablen, etwa zu Mobilitätsfaktoren und Anfahrtswegen der Patienten.

Trotz dieser vielfältigen Funktionen sind einige methodische Einschränkungen räumlich-epidemiologischer Analysen zu beachten, insbesondere bei der Nutzung aggregierter Daten. Geographische Einheiten können sehr unterschiedlich groß und unterschiedlich dicht besiedelt sein, während die statistischen Modelle häufig eine homogene Verteilung der Erkrankungen innerhalb dieser Einheiten annehmen [17]. Dadurch besteht die Gefahr, dass dünn besiedelte Gebiete entweder als Ausreißer erscheinen oder statistisch nicht signifikant werden – und somit unter Umständen fälschlicherweise vernachlässigt werden. Hinzu kommt das sog. „modifiable areal unit problem“ (MAUP): Die beobachteten geographischen Muster können sich je nach Wahl der räumlichen Aggregationsebene verändern, sodass Ergebnisse immer nur für die konkret analysierte Ebene gültig sind und nicht ohne Weiteres verallgemeinert werden dürfen [5, 13]. Eine zusätzliche Quelle möglicher Verzerrungen ist die Tatsache, dass Daten häufig auf einer anderen Ebene analysiert werden (z. B. regional), als sie ursprünglich erhoben wurden (z. B. individuell; [2]). Schließlich können fehlende oder unvollständige Daten zu einem Verlust statistischer Aussagekraft führen – ein Problem, das insbesondere bei seltenen Tumorentitäten zu berücksichtigen ist [6].

## Pathologie und Epidemiologie: Beziehung mit Zukunft

Die REDPath wurde von Beginn an nicht als klassisches statisches Werkzeug, sondern eher als dynamische Plattform konzipiert, die kontinuierlich weiterentwickelt und an neue lokale Anforderungen angepasst werden kann. Geplant ist in Kooperation mit dem Landeskrebsregister Rheinland-Pfalz, das im Institut für digitale Gesundheitsdaten Rheinland-Pfalz (IDG-RLP) verankert ist, um die vorliegenden pathologischen Routinedaten mit den auf gesetzlicher Basis erhobenen Krebsregisterdaten zu verknüpfen. Ziel ist es, Synergien mit bestehenden Strukturen zu schaffen, ohne parallele oder redundante Datenstrukturen aufzubauen. Ergänzend sollen die bereits eingebundenen Umweltdaten durch weitere Faktoren – etwa Luft- und Wasserqualität oder Feinstaubbelastung – erweitert werden, die vom Landesamt für Umwelt Rheinland-Pfalz bereitgestellt werden könnten. Langfristig ist zudem vorgesehen, klinische Daten des Universitären Centrums für Tumorerkrankungen (UCT) der Universitätsmedizin Mainz in die Plattform einzubinden. Parallel dazu wird an der Automatisierung zentraler Prozesse gearbeitet. Hierzu zählt die KI-gestützte Erfassung von ICD-O-3-Lokalisations- und Histologiecodes mithilfe sprachmodellbasierter Algorithmen, die den Kodierprozess effizienter und konsistenter gestalten sollen.

Dank seines modularen Aufbaus ist REDPath nicht auf onkologische Fragestellungen beschränkt, sondern lässt sich auf weitere medizinische Forschungsfelder übertragen, in denen räumliche Muster und Kontextfaktoren eine zentrale Rolle spielen, wie etwa in der Infektions- oder Umweltepidemiologie. Die zugrunde liegende Datenbankstruktur ermöglicht sowohl die Skalierung auf andere Krankheitsentitäten als auch die Einbeziehung zusätzlicher geographischer Regionen. Dies ist besonders relevant für Erkrankungen mit bekannter räumlicher Heterogenität, wie beispielsweise das Magenkarzinom [14]. An diesem Beispiel wird deutlich, dass nicht nur innerdeutsche Vergleiche, sondern perspektivisch auch Analysen im internationalen Kontext von Interesse sein könnten, sofern die Datenqualität und -verfügbarkeit ausreichend wären. Der Anschluss externer Datenquellen ließe sich über etablierte internationale Standards zur Kodierung und zum Austausch medizinischer Informationen wie HL7 FHIR oder SNOMED CT realisieren. Dies würde nicht nur die Integration in internationale Forschungsinfrastrukturen erleichtern, sondern auch Synergien mit bestehenden nationalen Initiativen wie der Medizininformatikinitiative (MII) oder der Nationalen Forschungsdateninfrastruktur für personenbezogene Gesundheitsdaten (NFDI4Health) schaffen. Über sichere, standardisierte Schnittstellen könnten so Datenquellen aus dem In- und Ausland eingebunden werden. Gerade in diesem Kontext ist ein transparentes und rechtssicheres Datenschutzkonzept unerlässlich.

Der Schutz personenbezogener Informationen ist ein zentrales Anliegen bei der Nutzung von Gesundheitsdaten für georäumliche Analysen. Im vorliegenden Projekt werden ausschließlich nach Art. 4 Abs. 5 EU-DSGVO pseudonymisierte und aggregierte Sekundärdaten verarbeitet, die keine Rückverfolgung auf Einzelpersonen ermöglichen. Es werden weder direkte noch indirekte Identifikationsmerkmale erhoben; die gespeicherten Daten beinhalten ausschließlich nicht-identifizierbare Informationen. Die Datenverarbeitung erfolgt auf räumlich- und passwortgeschützten Servern innerhalb des Instituts. Gemäß Art. 9 Abs. 3 EU-DSGVO liegt die Verantwortung für die Verarbeitung bei der Studienleitung, um eine ärztliche und technisch qualifizierte Handhabung sicherzustellen. Der Zugriff auf nicht-aggregierte Rohdaten ist auf die Studienleitung sowie auf eine autorisierte IT-Fachkraft beschränkt. Die ethische Unbedenklichkeit des Vorhabens wurde bereits im Rahmen eines positiven Ethikvotums durch die Ethikkommission Rheinland-Pfalz bestätigt (Antragsnummer: 2021-15741-retrospektiv, Datum des zustimmenden Votums: 13 April 2021). Zusätzlich schützt die räumliche Aggregation der Patientendaten – insbesondere bei seltenen Tumorentitäten – vor einer möglichen Reidentifizierbarkeit.

Um auch perspektivisch einer missbräuchlichen Nutzung durch wirtschaftliche oder politische Interessen vorzubeugen, ist die Einrichtung eines differenzierten Zugriffskonzepts nach dem Prinzip gestufter Analyseebenen („Zwiebelschalenprinzip“) vor Veröffentlichung des Programms vorgesehen. Während wissenschaftliche Institutionen und öffentliche Gesundheitseinrichtungen auf erweiterte Analysefunktionen zugreifen könnten, blieben diese für die allgemeine Öffentlichkeit technisch eingeschränkt – etwa durch IP-Filter oder rollenbasierte Nutzerrechte.

Insgesamt positioniert sich REDPath als Brücke zwischen Pathologie und Epidemiologie: als Werkzeug, das den enormen Datenschatz der pathologischen Routinediagnostik erstmals systematisch für räumlich-epidemiologische Analysen erschließt und zugleich eine Grundlage für eine zukunftsorientierte, patientenzentrierte Versorgung schafft.

## Fazit für die Praxis

- Pathologische Routinedaten bergen ein bislang unerschlossenes Potenzial für räumlich-epidemiologische Analysen in der Onkologie.
- Mit REDPath (räumlich-epidemiologische Datenanalyse von Pathologiedaten) steht erstmals ein web-basiertes Tool zur Verfügung, das Krankheitslasten und Versorgungssituationen interaktiv und ohne statistische Vorkenntnisse darstellt.
- Deskriptive und statistische Module machen regionale Muster, Risikofaktoren und Versorgungslücken sichtbar – insbesondere in Kombination mit Kontextinformationen wie Umwelt- und Demographiedaten.
- Für die Praxis bedeutet dies: REDPath kann für Versorgungs- und Ressourcenplanung sowie für die Entwicklung präventionsorientierter Strategien genutzt werden – von öffentlichen Gesundheitsdiensten, Krebsregistern, Krankenkassen und Forschungseinrichtungen.

## Data Availability

Kontext- und demografische Daten sowie die verwendeten Shapefiles zur räumlichen Darstellung wurden von der Statistikstelle des Bürgeramts der Stadt Mainz bezogen und können dort auf Anfrage eingesehen werden. Weitere Daten, einschließlich individueller Patientendaten und des vollständigen Quellcodes der entwickelten Software, können aus Gründen des Datenschutzes sowie aufgrund urheberrechtlicher und eigentumsrechtlicher Einschränkungen leider nicht bereitgestellt werden.
